# Risk factors for progressive kyphosis after percutaneous kyphoplasty in osteoporotic vertebral compression fracture

**DOI:** 10.1515/med-2024-1107

**Published:** 2024-12-09

**Authors:** Cong Jin, Lei He, Xi Chen, Jiewen Zheng, Wei He, Weiqi Han

**Affiliations:** Department of Orthopaedics, Shaoxing People’s Hospital, Shaoxing, Zhejiang, 312000, China; School of Medicine, Shaoxing University, Shaoxing, Zhejiang, 312000, China

**Keywords:** risk factors, kyphosis, kyphoplasty, osteoporotic fractures, nomograms

## Abstract

**Purpose:**

To investigate the risk factors associated with progressive kyphosis (PK) after percutaneous kyphoplasty (PKP) in osteoporotic vertebral compression fractures (OVCFs).

**Methods:**

A single-center retrospective study (January 2020 to December 2022) analyzed 129 OVCF patients treated with PKP. Patients were divided into a PK group and a non-progressive kyphosis group. Clinical and radiological data were compared, and univariate and multivariate regression analyses identified independent risk factors for PK. A nomogram was then developed to predict the risk factors for PK after PKP.

**Results:**

Of 129 patients, 47 (36.4%) experienced PK after PKP. Multivariate analysis identified independent risk factors for PK as preoperative kyphosis angle (OR = 1.26, *P* = 0.008), Type D magnetic resonance image (MRI) signal change on T2-weighted images (T2WI) (OR = 18.49, *P* = 0.003), black line signal (OR = 44.00, *P* < 0.001), intervertebral disc endplate complex (IDEC) injury (OR = 7.86, *P* = 0.021), and postoperative Oswestry Disability Index (ODI) score (OR = 1.18, *P* = 0.004). The nomogram, based on these factors, demonstrated strong discriminative performance (area under the curve = 0.953) and good calibration.

**Conclusions:**

Preoperative kyphosis angle, Type D MRI signal change on T2WI, black line signal, IDEC injury, and higher postoperative ODI score are independent risk factors for PK after PKP. A nomogram based on these factors accurately predicts PK risk.

## 1 Introduction

Osteoporotic vertebral compression fractures (OVCFs) are the most common fractures in patients with osteoporosis, capable of causing acute or chronic low back pain, kyphosis deformity, and cardiopulmonary dysfunction. These conditions can significantly impact patients’ quality of life, leading to high morbidity and mortality rates [1,2,3]. Percutaneous kyphoplasty (PKP) is considered an effective treatment for OVCFs, as it can restore the strength and stability of the vertebral body, providing significant pain relief [1,4]. Compared to conservative treatment, PKP significantly reduces the risk of complications such as pneumonia, deep vein thrombosis, and pressure sores caused by long-term bed rest, thereby lowering morbidity and mortality rates associated with OVCFs [3,5]. Papanastassiou et al. reported that compared to non-surgical treatment, PKP provided greater pain relief and fewer subsequent fractures in the treatment of OVCFs. PKP showed a slight advantage over percutaneous vertebroplasty (PVP) in terms of disability improvement and significantly outperformed PVP in enhancing quality of life [6]. Similarly, the study by Lange et al. demonstrated that patients with OVCFs who underwent surgical treatment had a higher overall survival rate compared to those receiving non-surgical treatment, with PKP patients potentially experiencing a survival advantage over those treated with PVP [7]. Svedbom et al. suggested that, in the United Kingdom, PKP may represent a cost-effective strategy for treating hospitalized patients with acute OVCFs compared to non-surgical treatment and PVP [8].

While PKP is associated with the risk of bone cement leakage and adjacent segment fractures, the literature reports incidences ranging from 5 to 40% for cement leakage and 5–15% for adjacent segment fractures within 1 year postoperatively [1–3,9]. Although previous literature has predominantly focused on the risk of bone cement leakage and adjacent segment fractures after PKP, few literature studies indicate a potential for further loss of vertebral height and recurrent kyphotic deformities after PKP. Additionally, it has been identified as a potential cause of postoperative residual pain [10,11]. Various factors may contribute to this, including the severity of OVCFs, residual postoperative kyphotic deformity, severity of osteoporosis, degeneration of adjacent intervertebral discs, and imbalance or weakness of paraspinal muscles [3,5,11–13].

To date, there have been no literature reports on the risk factors for progressive kyphosis (PK) after PKP for OVCFs. In the current study, clinical and radiological data from 129 OVCF patients who underwent PKP were retrospectively analyzed. The associations between various variables and PK were examined through univariate and multivariate regression analyses. Subsequently, a nomogram was developed to predict the risk factors for PK after PKP.

## 2 Materials and methods

### 2.1 Study design

From January 2020 to December 2022, a single-center retrospective study was conducted at Shaoxing People’s Hospital, retrospectively analyzing 129 patients with OVCFs treated with PKP. Based on whether PK occurred at the last follow-up, patients were divided into a PK group and a non-progressive kyphosis (NPK) group. Referring to previous literature, if the final follow-up kyphosis angle (KA) difference is greater than 10°, we define it as PK [14]. Clinical and radiological data of the two groups were compared, and a nomogram was developed to predict the risk factors for PK after PKP for OVCFs.

### 2.2 Participants

The inclusion criteria for this study consisted of patients aged over 60 years with single-segment OVCF caused by low-energy trauma, treated with PKP, preoperative bone mineral density (BMD) examination results of ≤−1.0 standard deviations (SD), and a minimum follow-up duration of 1 year. Exclusion criteria included secondary osteoporosis resulting from prolonged glucocorticoid use, endocrine disorders, and spinal infections; pathological fractures induced by primary or metastatic spinal tumors; and patients with severe cardiopulmonary insufficiency and coagulation disorders who were intolerant of surgery.

### 2.3 PKP

The same two surgeons performed all operations with the aid of C-arm fluoroscopy. The patient was in a prone position, with the chest and iliac crest elevated, and the abdomen suspended. Thereafter, the OVCF was reduced in the hyperextension position, and the needle entry point was marked under C-arm fluoroscopy guidance. Unilateral or bilateral transpedicular puncture was performed until the tip of the puncture cannula reached the anterior and middle thirds of the vertebral body in the lateral view. Subsequently, a working channel was created. After balloon expansion through the working channel implantation, bone cement (Heraeus, Germany) was gradually injected into the vertebral bodies. The patients could move freely with a soft brace 24 h postoperatively and received anti-osteoporosis treatment for 12 months. All PKP surgical instruments used during the procedure were manufactured by Canwell Company (Jinhua, China). Anti-osteoporotic management included daily oral administration of calcitriol at a dosage of 0.25 µg (Catalent Germany Eberbach GmbH, Eberbach, Germany) and calcium carbonate at 600 mg (Haleon, Suzhou, China). Zoledronic acid infusions (5 mg, Novartis, Basel, Switzerland) were initiated intravenously on the first postoperative day, with subsequent infusions administered annually for a 3-year course.

### 2.4 Baseline data outcomes

The baseline data for all patients were directly obtained from the hospital’s medical record information system, including gender, age, fracture location, height, weight, BMD, history of hypertension and diabetes, smoking status, alcohol consumption, and fracture type. BMD was measured using dual-energy X-ray absorptiometry, with the measured BMD being the average value from L1 to L4, excluding the fractured vertebra.

### 2.5 Radiographic outcomes

Radiological evaluation parameters, encompassing vertebral height loss (VHL) and KA, were assessed using electronic measurement tools integrated into the Picture Archiving and Communication System (PACS V3.0, Zhejiang Rad Information Technology Company, Hangzhou, China). These measurements were conducted by two radiologists, and the results were subsequently averaged. To minimize evaluation bias, all assessments were conducted in a blinded manner. The radiologists performing the evaluations were unaware of the clinical details and treatment assignments of the patients, ensuring objectivity in the assessments.

**Figure 1:**
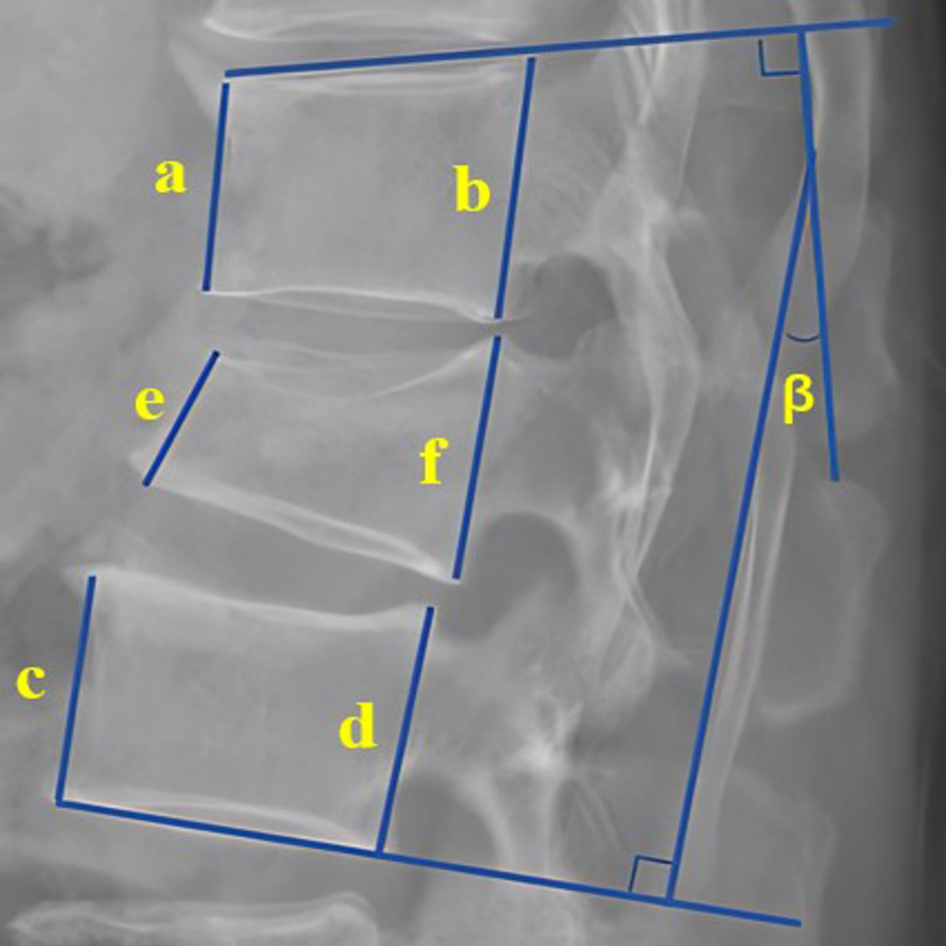
Measurement method for VHL and KA. (1) The KA (*β*) was measured as the angle between the superior endplate of the vertebra above and the inferior endplate of the vertebra below on lateral radiography. (2) (a) Anterior height of the cranial vertebrae, (b) posterior height of the cranial vertebrae, (c) anterior height of the caudal vertebrae, (d) posterior height of the caudal vertebrae, (e) anterior height of the fractured vertebrae, (f) posterior height of the fractured vertebrae. VHL = \[\left[\frac{\frac{(a+b+c+d)}{4}-\frac{(e+f)}{2}}{\frac{(a+b+c+d)}{4}}\right]\times 100 \% ]\] .

The VHL was measured on lateral X-rays preoperatively, postoperatively, and at the last follow-up. The methodology for VHL measurement is elucidated in Figure 1. The postoperative VHL difference denotes the subtraction of preoperative VHL from postoperative VHL. The VHL improvement rate is calculated as the postoperative VHL difference divided by preoperative VHL, multiplied by 100%. Furthermore, the VHL difference at the last follow-up is defined as the subtraction of VHL at the last follow-up from postoperative VHL.

The KA was measured preoperatively, postoperatively, and at the last follow-up as the angle between the superior endplate of the vertebra above and the inferior endplate of the vertebra below on lateral radiography (Figure 1). Postoperative KA difference is determined by subtracting preoperative KA from postoperative KA. The KA improvement rate is computed as the postoperative KA difference divided by preoperative KA, multiplied by 100%. Similarly, the KA difference at the last follow-up is defined as the subtraction of KA at the last follow-up from postoperative KA. If the KA difference at the last follow-up exceeded 10°, it was classified as PK.

### 2.6 Magnetic resonance image (MRI) evaluation

**Figure 2:**
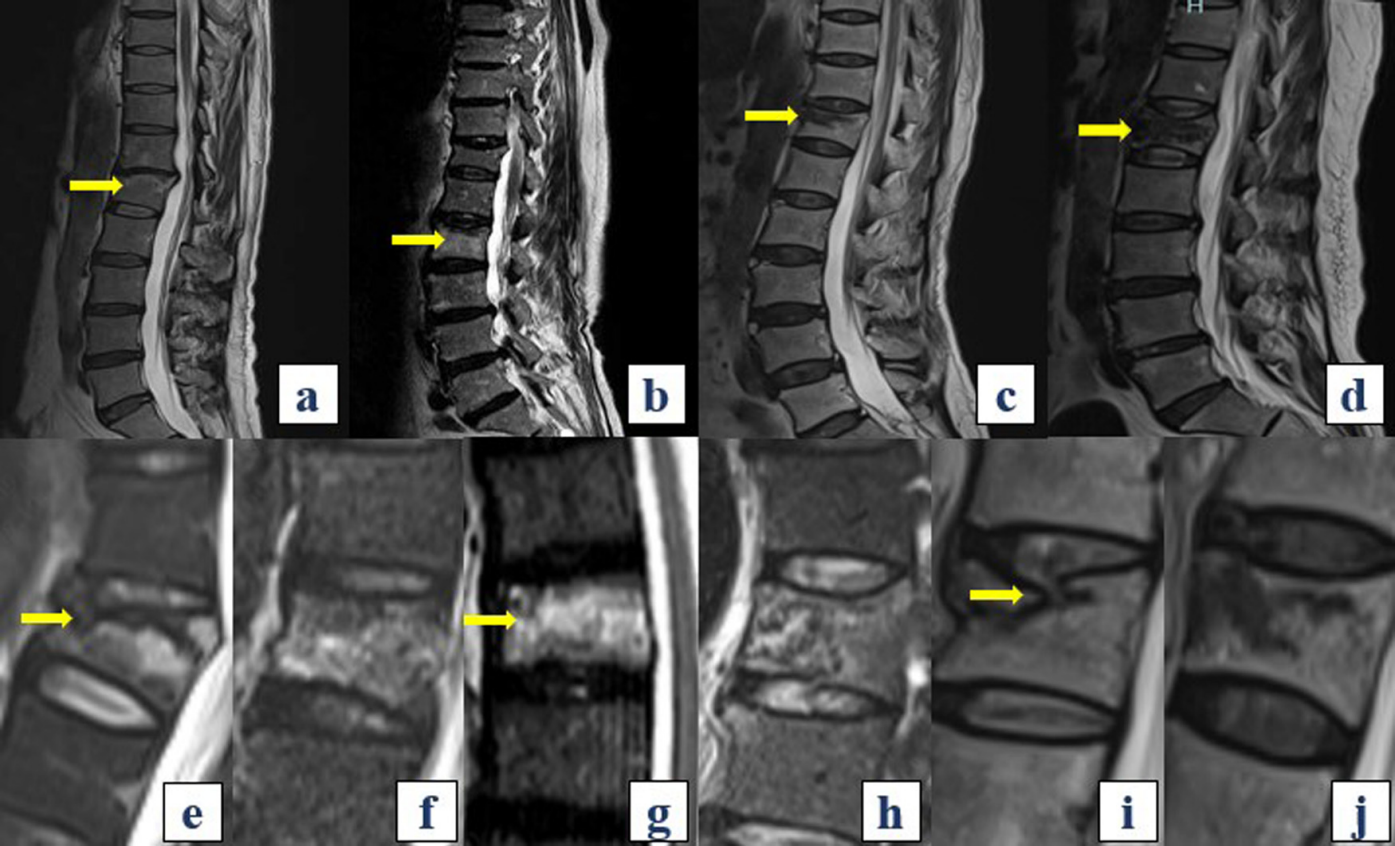
Fracture assessment on magnetic resonance imaging. (a)–(d) Subtyping based on MRI signal changes on T2WI. (a) Type A – confined high signal change (yellow arrow), (b) Type B – diffuse high signal change (yellow arrow), (c) Type C – confined low signal change (yellow arrow), and (d) Type D – diffuse low signal change (yellow arrow). (e) and (f) Typical black line signal change on STIR images. Black line signal is defined as a black line exceeding half of the anteroposterior diameter of the vertebral body on STIR images. (e) Typical black line signal (yellow arrow) and (f) non-black line signal. (g) and (h) Typical homogeneous high signal change on STIR images. Homogeneous high signal is defined as an area of homogeneous high signal exceeding half of the vertebral body area on STIR images. (g) Typical homogeneous high signal (yellow arrow) and (h) non-homogeneous high signal. (i) and (j) Assessment of IDEC integrity on T2WI. (i) Injury to the IDEC above the fractured vertebra (yellow arrow). (j) Intact IDEC both above and below the injured vertebra. T2WI: T2-weighted images; STIR: short tau inversion recovery; IDEC: intervertebral disc endplate complex.

Thoracolumbar MRI examinations were undertaken for all patients preoperatively using a 1.5 T MRI device (Siemens, Germany) equipped with a spine coil. The examination protocol comprised T1-weighted spin echo (SE) sagittal, T2-weighted SE axial, T2-weighted SE sagittal, and short tau inversion recovery (STIR) sagittal sections. Diagnosis of recent OVCF was established based on the presence of a hypointense signal on T1-weighted images. Furthermore, classifications of MRI signal changes on T2-weighted images (T2WI) [15] were obtained (Figure 2a–d). Data pertaining to black line signal (Figure 2e and f) and homogeneous high signal (Figure 2g and h) were derived from STIR images [16], while evaluation of the integrity of the intervertebral disc endplate complex (IDEC) was conducted on T2WI [17,18] (Figure 2i and j).

### 2.7 Clinical outcomes

Clinical outcomes were evaluated through the utilization of a visual analog scale (VAS) [19] and the Oswestry Disability Index (ODI) [20]. Measurement of pain on the VAS was performed, with zero representing the absence of pain and ten signifying intolerable pain. Assessment of pain levels occurred at preoperative, postoperative, and last follow-up time points. Self-assessment of disability using the ODI was conducted concurrently at the preoperative, postoperative, and last follow-up stages.

### 2.8 Operative outcomes

**Figure 3:**
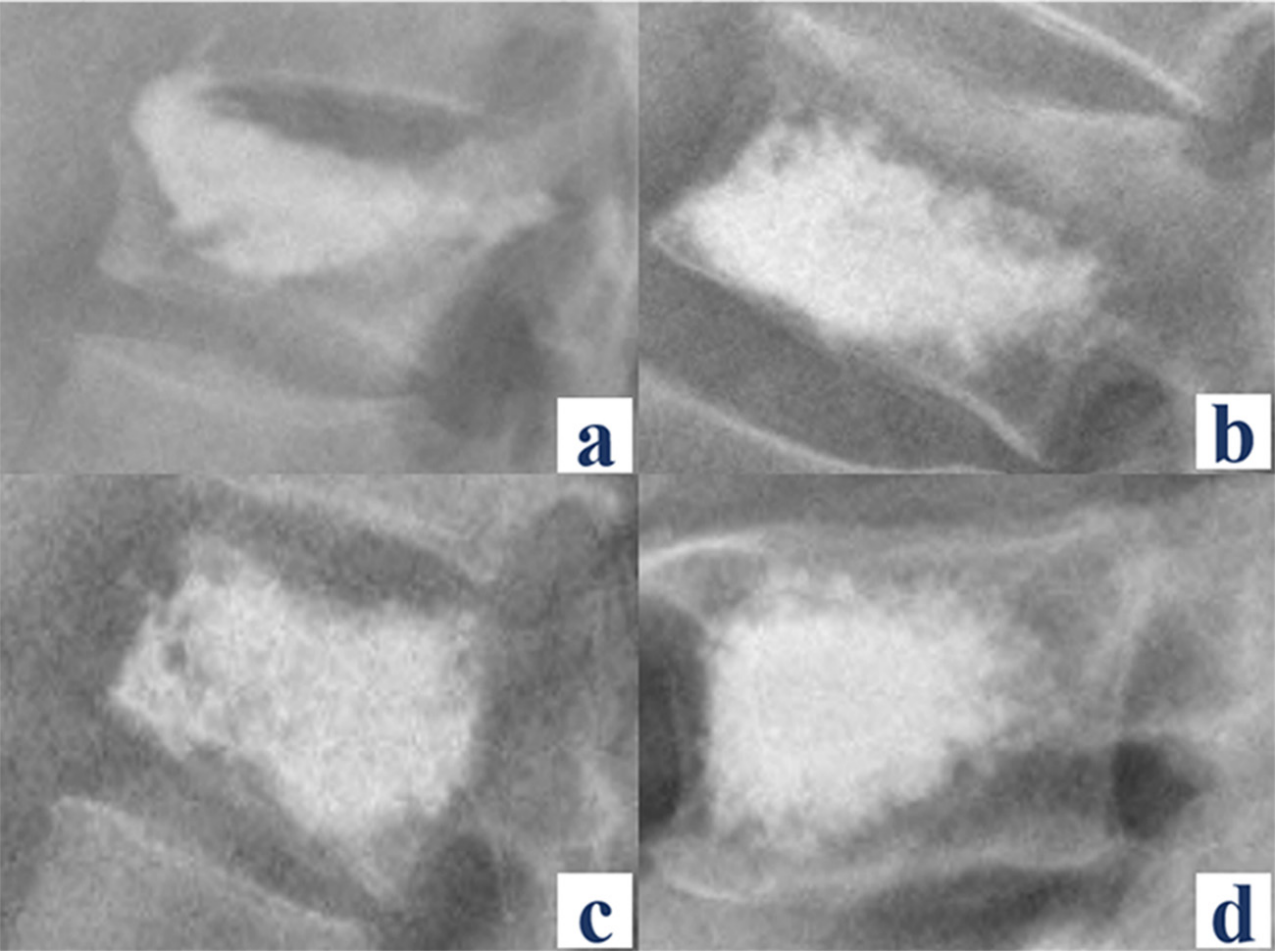
Classification of bone cement form on lateral radiography. (a) Type A – bone cement contacts only the superior endplate of the vertebral body. (b) Type B – bone cement extends only to the inferior endplate of the vertebral body. (c) Type C – bone cement contacts both the superior and inferior endplates of the vertebral body simultaneously. (d) Type D – bone cement does not contact either the superior or the inferior endplate of the vertebral body simultaneously.

Operative data, encompassing operative time, unilateral or bilateral puncture, and volume of injected bone cement, were directly extracted from the surgical records in the hospital’s electronic medical record system. In alignment with previous literature [21], the assessment of bone cement form classifications was conducted based on postoperative lateral X-rays (Figure 3). Furthermore, postoperative X-rays in both anteroposterior and lateral views were employed to ascertain the presence of bone cement leakage and determine its location.

### 2.9 Statistical analysis

Statistical analysis was conducted using SPSS (version 19.0; SPSS Inc., Chicago, IL, USA) on the Windows platform. The comparison of gender, history of hypertension, fracture type, black line signal, homogeneous high signal, IDEC injury, and cement leakage between the two groups was executed through the utilization of the Chi-squared test. Yates’ correction was implemented to assess fracture location, history of diabetes, smoking status, alcohol consumption, MRI signal change on T2WI, unilateral or bilateral puncture, bone cement form, and leakage site between the groups.

Evaluation of age, height, weight, postoperative VHL, and postoperative KA difference was carried out through independent samples *t*-tests, with the confirmation of normal distribution by the Shapiro–Wilk normality test and assessment of variance homogeneity by Levene’s test. Welch *t*-tests were applied to compare VHL at the last follow-up, VHL difference at the last follow-up, preoperative KA, postoperative KA, and KA at the last follow-up between the groups.

Body mass index (BMI), BMD, postoperative VHL difference, VHL improvement rate, KA improvement rate, KA difference at the last follow-up, operative time, and bone cement volume between the groups were assessed using Wilcoxon tests. Two-way mixed ANOVA tests were employed to analyze the VAS and ODI scores. Furthermore, the relationship between PK and independent variables was modeled using logistic regression. The significance level was set at 0.05.


**Informed consent:** Informed consent was obtained from all patients.
**Ethical approval:** The authors confirm that the study was performed in accordance with the Declaration of Helsinki. The study was approved by the Ethics Committee of the Shaoxing People’s Hospital, reference number NO202404101.

## 3 Results

### 3.1 Baseline data

A total of 129 patients were enrolled in this study, with 47 cases (36.4%) assigned to the PK group and 82 cases (63.6%) to the NPK group. In terms of gender, age, height, weight, BMI, BMD, history of hypertension, history of diabetes, smoking, and alcohol consumption, no significant statistical differences were observed between the two groups. However, there were notable statistical differences in fracture location and fracture type (*P* < 0.001) (Table 1).

**Table 1:** Comparison of baseline data between the two groups

Characteristics	NPK group	PK group	*P* value
*n*	82	47	
Gender, *n* (%)			0.204
Male	22 (17.1%)	8 (6.2%)	
Female	60 (46.5%)	39 (30.2%)	
Age, mean ± SD	69.67 ± 8.42	71.57 ± 7.50	0.201
Fracture location, *n* (%)			<0.001
TL	52 (40.3%)	47 (36.4%)	
L	22 (17.1%)	0 (0%)	
T	8 (6.2%)	0 (0%)	
Height (m), mean ± SD	1.60 ± 0.07	1.58 ± 0.08	0.136
Weight (kg), mean ± SD	59.32 ± 9.03	56.71 ± 10.27	0.136
BMI (kg/m^2^), median (IQR)	22.77 (20.85, 24.12)	22.22 (20.01, 24.19)	0.411
BMD, median (IQR)	−2.95 (−3.78, −2.03)	−3.1 (−4.00, −2.20)	0.185
Hypertension, *n* (%)			0.143
Yes	31 (24%)	24 (18.6%)	
No	51 (39.5%)	23 (17.8%)	
Diabetes, *n* (%)			0.284
No	76 (58.9%)	40 (31%)	
Yes	6 (4.7%)	7 (5.4%)	
Smoking, *n* (%)			1.000
No	80 (62%)	46 (35.7%)	
Yes	2 (1.6%)	1 (0.8%)	
Alcohol, *n* (%)			0.381
No	78 (60.5%)	42 (32.6%)	
Yes	4 (3.1%)	5 (3.9%)	
Fracture type, *n* (%)			<0.001
Compression fracture	78 (60.5%)	30 (23.3%)	
Burst fracture	4 (3.1%)	17 (13.2%)	

NPK: non-progressive kyphosis; PK: progressive kyphosis; BMI: body mass index; BMD: bone mineral density; SD: standard deviation; IQR: interquartile range.

### 3.2 Radiographic outcomes

The preoperative VHL, postoperative VHL difference, VHL improvement rate, VHL at the last follow-up, and VHL difference at the last follow-up in the NPK group were found to be significantly lower than those observed in the PK group (*P* < 0.05). However, no significant statistical difference was identified between the postoperative VHL of the two groups.

Similarly, the preoperative KA, postoperative KA, postoperative KA difference, KA improvement rate, KA at the last follow-up, and KA difference at the last follow-up in the NPK group were significantly lower than those in the PK group, indicating significant statistical differences (*P* < 0.05).

Furthermore, the NPK group exhibited statistical differences compared to the PK group in terms of MRI signal change on T2WI (*P* < 0.001). The NPK group also demonstrated significantly lower proportions of black line signal and IDEC injury than the PK group, with statistical significance (*P* < 0.001). Additionally, the NPK group displayed a significantly higher proportion of homogeneous high signal compared to the PK group, with statistically significant differences (*P* < 0.001) (Table 2).

**Table 2:** Comparison of radiographic data between the two groups

Characteristics	NPK group	PK group	*P* value
Preoperative VHL, median (IQR)	11.65 (7.10, 17.88)	17.90 (12.45, 28.20)	<0.001
Postoperative VHL, mean ± SD	10.70 ± 8.03	12.89 ± 10.51	0.188
Postoperative VHL difference, median (IQR)	1.35 (−2.20, 5.73)	5.9 (−1.05, 12.65)	0.006
VHL improvement rate, median (IQR)	10.83 (−13.16, 48.59)	32.80 (−0.77, 64.06)	0.047
VHL at the last follow-up, mean ± SD	12.73 ± 8.121	18.84 ± 11.05	0.001
VHL difference at the last follow-up, mean ± SD	2.03 ± 5.09	5.95 ± 6.86	0.001
Preoperative KA, mean ± SD	7.54 ± 16.64	22.86 ± 12.34	<0.001
Postoperative KA, mean ± SD	6.68 ± 17.16	18.76 ± 10.52	<0.001
Postoperative KA difference, mean ± SD	0.86 ± 4.68	4.10 ± 4.63	<0.001
KA improvement rate, median (IQR)	1.72 (−16.01, 22.00)	19.62 (4.25, 26.97)	0.003
KA at the last follow-up, mean ± SD	7.27 ± 17.14	30.70 ± 10.49	<0.001
KA difference at the last follow-up, median (IQR)	0.45 (−1.30, 2.88)	11.20 (10.60, 12.15)	<0.001
MRI signal changes on T2WI, *n* (%)			<0.001
A	7 (5.4%)	1 (0.8%)	
B	3 (2.3%)	1 (0.8%)	
C	59 (45.7%)	20 (15.5%)	
D	13 (10.1%)	25 (19.4%)	
Black line signal, *n* (%)			<0.001
No	75 (58.1%)	20 (15.5%)	
Yes	7 (5.4%)	27 (20.9%)	
Homogenous high signal, *n* (%)			<0.001
No	48 (37.2%)	41 (31.8%)	
Yes	34 (26.4%)	6 (4.7%)	
IDEC injury, *n* (%)			<0.001
No	67 (51.9%)	24 (18.6%)	
Yes	15 (11.6%)	23 (17.8%)	

NPK: non-progressive kyphosis; PK: progressive kyphosis; VHL: vertebral height loss; KA: kyphosis angle; SD: standard deviation; IQR: interquartile range; T2WI: T2-weighted images; IDEC: intervertebral disc endplate complex.

### 3.3 Clinical outcomes

**Figure 4:**
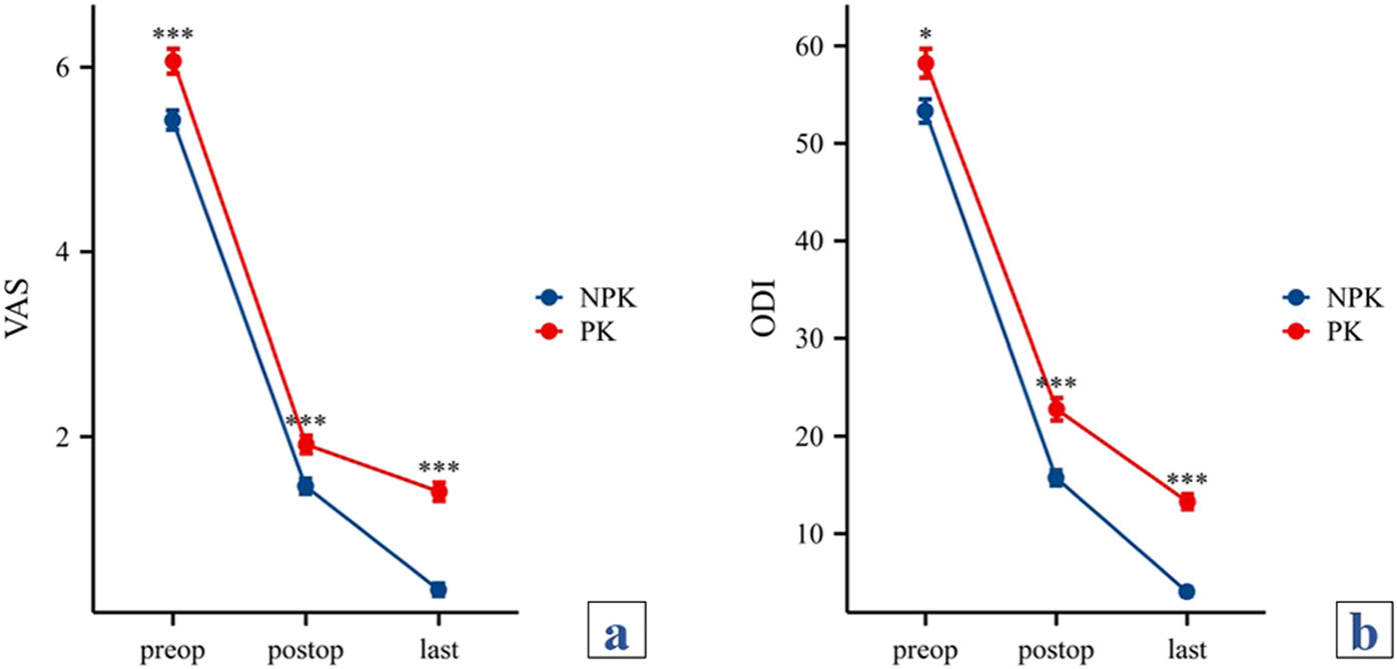
(a) Comparison of VAS scores between the two groups. (b) Comparison of ODI scores between the two groups. *P < 0.05, compared with NPK group; ***P < 0.001, compared with NPK group. VAS: visual analog scale; ODI: Oswestry Disability Index; NPK: non-progressive kyphosis; PK: progressive kyphosis.

Preoperatively, postoperatively, and at the last follow-up, VAS scores of the NPK group were found to be significantly lower than those of the PK group, indicating statistical differences (*P* < 0.001). Additionally, preoperatively, postoperatively, and at the last follow-up, ODI scores of the NPK group were likewise determined to be significantly lower than those of the PK group (*P* < 0.05) (Figure 4).

### 3.4 Operative outcomes

Operative time, bone cement volume, unilateral or bilateral procedures, bone cement forms, proportion of bone cement leakage, and leakage site were found to be comparable between the two groups (*P* > 0.05) (Table 3).

**Table 3:** Comparison of operative data between the two groups

Characteristics	NPK	PK	*P* value
Operative time (min), median (IQR)	32 (25.5, 40)	30 (25, 39.5)	0.424
Bone cement volume (mL), median (IQR)	6 (6, 6)	6 (5, 6)	0.278
Unilateral or bilateral, *n* (%)			1.000
Bilateral	79 (61.2%)	45 (34.9%)	
Unilateral	3 (2.3%)	2 (1.6%)	
Bone cement form, *n* (%)			0.267
A	7 (5.4%)	2 (1.6%)	
B	22 (17.1%)	7 (5.4%)	
C	46 (35.7%)	34 (26.4%)	
D	7 (5.4%)	4 (3.1%)	
Cement leakage, *n* (%)			0.184
No	50 (38.8%)	23 (17.8%)	
Yes	32 (24.8%)	24 (18.6%)	
Leakage site, *n* (%)			0.597
No leakage	50 (38.8%)	23 (17.8%)	
Intervertebral space	6 (4.7%)	6 (4.7%)	
Lateral vertebrae	10 (7.8%)	9 (7%)	
Anterior vertebrae	8 (6.2%)	5 (3.9%)	
Basivertebral foramen	8 (6.2%)	4 (3.1%)	

NPK: non-progressive kyphosis; PK: progressive kyphosis; IQR: interquartile range.

### 3.5 Risk factors for PK after PKP

In the univariate regression analysis, risk factors for PK after PKP included burst fracture, preoperative VHL, preoperative KA, postoperative KA, Type D MRI signal change on T2WI, black line signal, non-homogeneous high signal, and IDEC injury. Subsequently, in the multivariate analysis, the independent risk factors were found to be preoperative KA (OR = 1.26, 95% CI 1.06–1.48, *P* = 0.008), Type D MRI signal change on T2WI (OR = 18.49, 95% CI 2.73–125.15, *P* = 0.003), black line signal (OR = 44.00, 95% CI 5.22–370.93, *P* < 0.001), IDEC injury (OR = 7.86, 95% CI 1.36–45.39, *P* = 0.021), and postoperative ODI score (OR = 1.18, 95% CI 1.05–1.33, *P* = 0.004) (Table 4).

**Table 4:** Univariate and multivariate analysis of risk factors for PK after PKP

Characteristics	Univariate analysis		Multivariate analysis	
	Odds ratio (95% CI)	*P* value	Odds ratio (95% CI)	*P* value
Fracture type				
Compression fracture	Reference		Reference	
Burst fracture	11.05 (3.44–35.52)	<0.001	2.19 (0.21–22.80)	0.512
Preoperative VHL	1.06 (1.02–1.09)	0.003	0.93 (0.85–1.02)	0.111
Postoperative VHL	1.03 (0.99–1.07)	0.189		
VHL improvement rate	1.001 (0.999–1.003)	0.539		
Preoperative KA	1.08 (1.04–1.12)	<0.001	1.26 (1.06–1.48)	0.008
Postoperative KA	1.06 (1.03–1.09)	<0.001	0.92 (0.80–1.06)	0.234
KA improvement rate	1.000 (0.998–1.002)	0.937		
MRI signal change on T2WI				
A	0.42 (0.05–3.64)	0.432	1.89 (0.07–53.41)	0.708
B	0.98 (0.10–10.00)	0.989	5.99 (0.24–152.54)	0.278
C	Reference		Reference	
D	5.67 (2.45–13.15)	<0.001	18.49 (2.73–125.15)	0.003
Black line signal				
No	Reference		Reference	
Yes	14.46 (5.50–38.03)	<0.001	44.00 (5.22–370.93)	<0.001
Homogenous high signal				
No	Reference		Reference	
Yes	0.21 (0.80–0.54)	0.001	0.28 (0.04–1.77)	0.175
IDEC injury				
No	Reference		Reference	
Yes	4.28 (1.92–9.53)	<0.001	7.86 (1.36–45.39)	0.021
Preoperative VAS score	2.06 (1.36–3.11)	<0.001	1.01 (0.30–3.35)	0.986
Preoperative ODI score	1.04 (1.01–1.08)	0.015	0.94 (0.84–1.04)	0.230
Postoperative ODI score	1.13 (1.07–1.20)	<0.001	1.18 (1.05–1.33)	0.004

NPK: non-progressive kyphosis; PK: progressive kyphosis; VHL: vertebral height loss; KA: kyphosis angle; T2WI: T2-weighted images; IDEC: intervertebral disc endplate complex; VAS: visual analog scale; ODI: Oswestry Disability Index.

### 3.6 Nomogram for risk factors of PK after PKP

**Figure 5:**
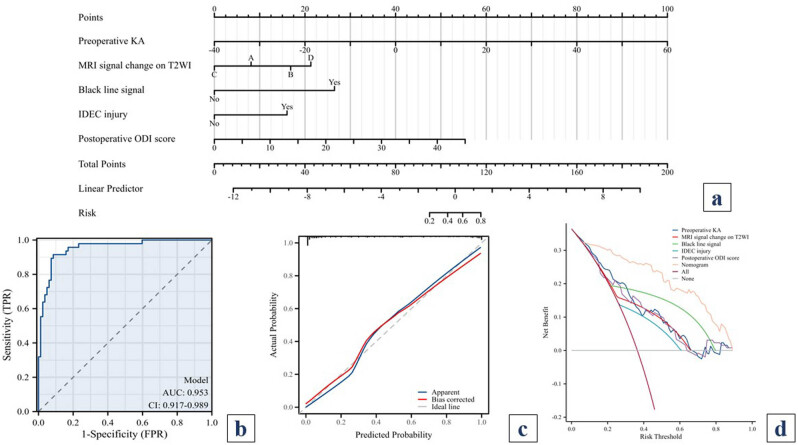
Nomogram for risk factors of PK after PKP. (a) Nomogram, (b) receiver operating characteristic curve, (c) calibration curve, and (d) DCA curve.

**Figure 6:**
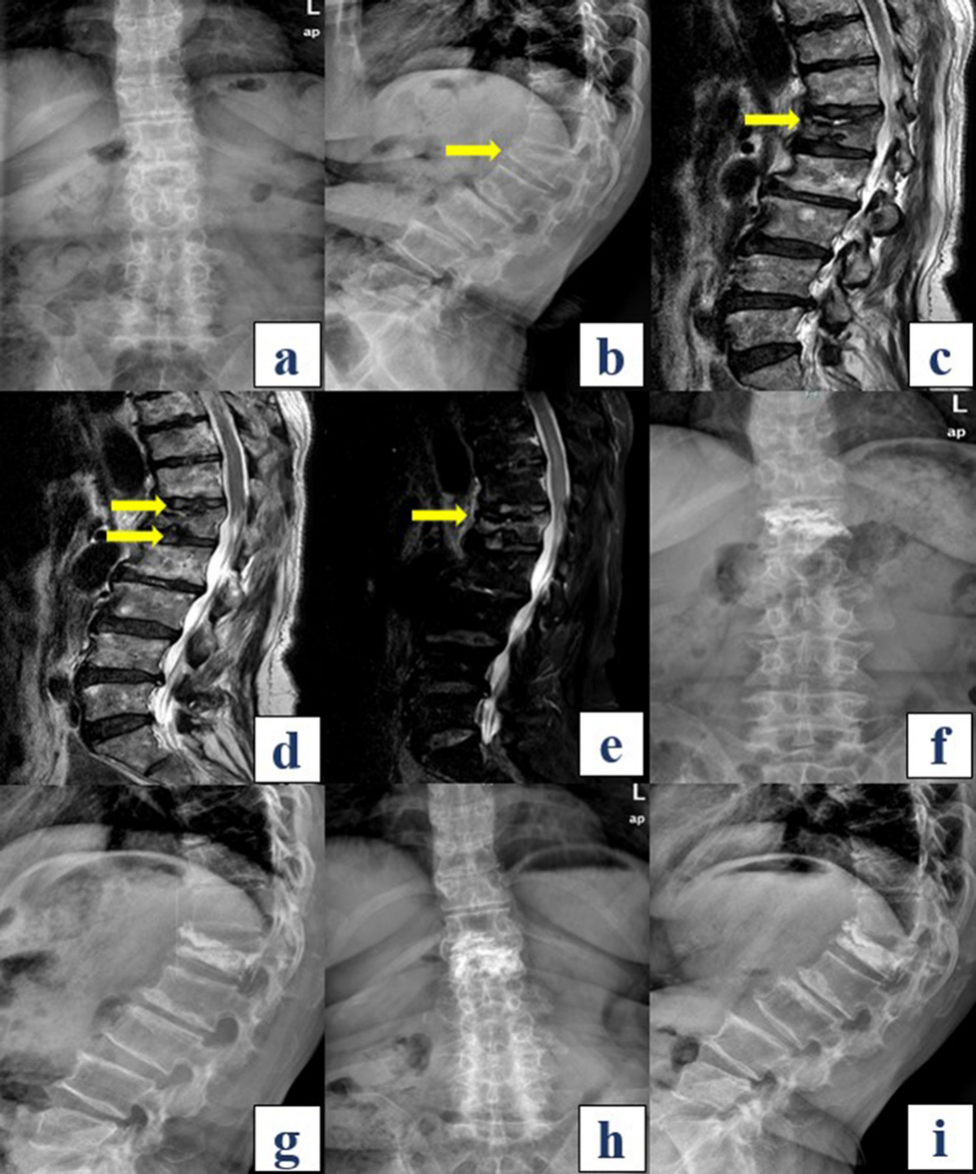
Presentation of a typical case in the PK group. A 65-year-old female with an L1 compression fracture due to a fall. (a) and (b) Preoperative X-ray indicating a compression fracture at L1, with a preoperative KA of 39.9° (yellow arrow). (c) Preoperative T2-weighted MRI showing diffuse low signal (Type D, indicated by the yellow arrow). (d) Preoperative MRI suggesting injury to the IDEC both above and below the injured vertebra (yellow arrow). (e) Preoperative STIR MRI indicating a black line signal (yellow arrow). (f) and (g) Postoperative X-ray showing a significant correction in the KA to 33.1°, well-distributed bone cement within the vertebral body, and no apparent leakage. (h) and (i) Last follow-up X-ray revealing an increase in KA to 45.0°, with a difference of 11.9° from the postoperative KA, indicating a significant progression of kyphosis. KA: kyphosis angle; STIR: short tau inversion recovery.

A nomogram was constructed based on the multivariate analysis of independent risk factors (Figure 5a). The discriminative ability of the model was assessed by plotting the receiver operating characteristic curve (Figure 5b). The area under the curve was calculated to be 0.953 (CI: 0.917–0.989), indicating robust discriminative performance. Calibration curves illustrated the congruence between predicted outcomes and actual results (Figure 5c). The decision curve analysis (DCA) curve was employed to predict the risk of PK using the five risk factors or their combination. The results demonstrated a significant increase in net benefit with the amalgamated nomogram model (Figure 5d). Overall, the model demonstrated feasibility and suitability for prediction (Figure 6).

## 4 Discussion

PK after PKP is a not uncommon occurrence in clinical practice, and limited literature exists exploring its potential risk factors. In this retrospective study, an investigation into the possible risk factors for PK following PKP was undertaken, and a nomogram for risk prediction was developed. Among the notable findings, larger preoperative KA, Type D MRI signal change on T2WI, black line signal, IDEC injury, and higher preoperative ODI score were identified as independent risk factors for PK after PKP. The nomogram, based on these factors, predicts the risk of PK, providing valuable insights for clinicians when deciding on treatment strategies.

Wang et al. reported that a greater correction of vertebral height and kyphotic angles constitutes a risk factor for the recurrence of kyphotic deformity after PKP [14]. In our investigation, statistical differences in the improvement rates of VHL and KA were observed between the NPK and PK groups. However, no statistical difference was found in univariate analysis. Nevertheless, a larger preoperative KA was identified as a risk factor for PK following PKP. Chou et al. documented 14 cases of PK after PVP, with an average preoperative KA of 23.67° [22]. In this study, the preoperative KA in the PK group was measured at 22.86°, demonstrating comparability. The plausible explanation is that a larger KA contributes to diminished spinal stability, thereby elevating the risk of PK in subsequent stages [23,24]. Additionally, this study revealed a significantly greater preoperative KA in the PK group (22.86 ± 12.34) compared to the NPK group (7.54 ± 16.64), and the postoperative KA in the PK group (18.76 ± 10.52) remained significantly higher than in the NPK group (6.68 ± 17.16). This observation implies that the kyphotic deformity in patients of the PK group was not fully rectified by PKP, and the residual deformity may serve as a potential factor for the progression of postoperative kyphotic deformity.

Tsujio et al. initially reported the classification of MRI signals on T2WI of OVCFs. They found that patients with diffuse low signal (Type D) were at a higher risk of vertebral collapse compared to those with a high signal [15]. Referring to their classification method, we also classified patients based on MRI signals on T2WI and identified Type D signal change as a risk factor for PK after PKP. It is hypothesized that a more extensive area of low signal on MRI may signify more severe fracture damage, indicating a greater disruption of trabecular bone. Conversely, a high signal may denote localized edema, suggesting relatively less severe damage to trabecular bone.

Characteristic signals, including homogeneous high signal and black line signal, are discernible on MRI STIR images in OVCFs, as initially documented by Omi et al. [16]. Omi also highlighted that non-homogeneous high signal and black line signal are deemed high-risk factors for nonunion of fractures [16]. In this study, the proportion of non-homogeneous high signal in the PK group was notably higher than that in the NPK group. While this distinction exhibited statistical significance in univariate analysis, it did not maintain significance in multivariate analysis. The black line signal was recognized as an independent risk factor for PK after PKP (OR = 44.00). This suggests that, in clinical practice, OVCF patients displaying black line signals should receive heightened attention, prompting a more proactive approach to treatment.

Previous literature has reported that the IDEC injury is a risk factor for the recurrence of kyphosis after PKP surgery [14]. In this study, we also found that IDEC injury is a risk factor for PK after PKP (OR = 7.86). The possible reason is that IDEC injury leads to long-term degeneration of the intervertebral disc, loss of intervertebral space height, and subsequently exacerbates kyphotic deformity.

In this study, we established a nomogram model and each variable included in the nomogram is a relatively easily accessible factor. By calculating the score for each of these five factors, orthopedic surgeons can easily estimate the risk of PK after PKP in patients with OVCFs. For patients assessed with lower risk, PKP remains a safe and effective treatment option. However, for those assessed with higher risk, recommendations may include prolonged bed rest, proactive osteoporosis treatment, and even adjunctive internal fixation.

This study has several limitations that should be acknowledged. The retrospective nature of this single-center analysis may introduce biases in case selection and data completeness, limiting the generalizability of the findings. Additionally, the inclusion criterion of a BMD result of ≤−1.0 SD may affect the applicability of the conclusions, as the World Health Organization’s 1994 criteria require a *T*-score ≤−2.5 SD for osteoporosis diagnosis, while the 2017 Chinese Guidelines [25] allow for diagnosis based on fragility fractures or T-scores between −2.5 and −1.0 with fractures. Furthermore, the nomogram was developed using data from a single institution, which restricts its applicability across different clinical settings. One parameter was assessed postoperatively, potentially compromising its accuracy in predicting the risk of PK after PKP. Finally, the exclusion of parathyroid hormone (PTH) as a treatment option limits the study. Although PTH is recognized as an effective first-line treatment due to its bone anabolic properties, its use was limited by cost and availability constraints within our patient population. Future research should explore PTH’s role in preventing postoperative complications, such as recurrent fractures and PK, after PKP, as this could enhance treatment strategies for high-risk patients.

## 5 Conclusions

A larger preoperative KA, Type D MRI signal change on T2WI, black line signal, IDEC injury, and higher preoperative ODI scores were independent risk factors for PK after PKP. An accurate nomogram was developed based on these factors.

## Abbreviations

BMD: bone mineral density
BMI: body mass index
DCA: decision curve analysis
IDEC: intervertebral disc endplate complex
IQR: interquartile range
KA: kyphosis angle
MRI: magnetic resonance image
NPK: non-progressive kyphosis
ODI: Oswestry Disability Index
OVCFs: osteoporotic vertebral compression fractures
PK: progressive kyphosis
PKP: percutaneous kyphoplasty
PTH: parathyroid hormone
PVP: percutaneous vertebroplasty
SD: standard deviations
SE: spin echo
STIR: short tau inversion recovery
T2WI: T2-weighted images
VAS: visual analog scale
VHL: vertebral height loss

## References

[1] Zhao G, Liu X, Li F. Balloon kyphoplasty versus percutaneous vertebroplasty for treatment of osteoporotic vertebral compression fractures (OVCFs). Osteoporos Int. 2016;27(9):2823–34. 10.1007/s00198-016-3610-y 27121344

[2] Lee JK, Jeong HW, Joo IH, Ko YI, Kang CN. Percutaneous balloon kyphoplasty for the treatment of very severe osteoporotic vertebral compression fractures: a case–control study. Spine J. 2018;18(6):962–9. 10.1016/j.spinee.2017.10.006 29055740

[3] Zhu RS, Kan SL, Ning GZ, Chen LX, Cao ZG, Jiang ZH, et al. Which is the best treatment of osteoporotic vertebral compression fractures: balloon kyphoplasty, percutaneous vertebroplasty, or non-surgical treatment? A Bayesian network meta-analysis. Osteoporos Int. 2019;30(2):287–98. 10.1007/s00198-018-4804-2 30635698

[4] Jindal V, Binyala S, Kohli SS. Balloon kyphoplasty versus percutaneous vertebroplasty for osteoporotic vertebral body compression fractures: clinical and radiological outcomes. Spine J. 2023;23(4):579–84. 10.1016/j.spinee.2022.11.015 36481681

[5] Muratore M, Ferrera A, Masse A, Bistolfi A. Osteoporotic vertebral fractures: predictive factors for conservative treatment failure. A systematic review. Eur Spine J. 2018;27(10):2565–76. 10.1007/s00586-017-5340-z 29030703

[6] Papanastassiou ID, Phillips FM, Van Meirhaeghe J, Berenson JR, Andersson GB, Chung G, et al. Comparing effects of kyphoplasty, vertebroplasty, and non-surgical management in a systematic review of randomized and non-randomized controlled studies. Eur Spine J. 2012;21(9):1826–43. 10.1007/s00586-012-2314-z PMC3459114 22543412

[7] Lange A, Kasperk C, Alvares L, Sauermann S, Braun S. Survival and cost comparison of kyphoplasty and percutaneous vertebroplasty using German claims data. Spine (Phila Pa 1976). 2014;39(4):318–26. 10.1097/BRS.0000000000000135 24299715

[8] Svedbom A, Alvares L, Cooper C, Marsh D, Strom O. Balloon kyphoplasty compared to vertebroplasty and nonsurgical management in patients hospitalised with acute osteoporotic vertebral compression fracture: a UK cost-effectiveness analysis. Osteoporos Int. 2013;24(1):355–67. 10.1007/s00198-012-2102-y PMC3691631 22890362

[9] Shi X, Li P, Li J, Bao C, Xiang J, Lu Y. Comparative evaluation of an innovative deflectable percutaneous kyphoplasty versus conventional bilateral percutaneous kyphoplasty for osteoporotic vertebral compression fractures: a prospective, randomized and controlled trial. Spine J. 2023;23(4):585–98. 10.1016/j.spinee.2022.12.012 36563860

[10] Li Y, Yue J, Huang M, Lin J, Huang C, Chen J, et al. Risk factors for postoperative residual back pain after percutaneous kyphoplasty for osteoporotic vertebral compression fractures. Eur Spine J. 2020;29(10):2568–75. 10.1007/s00586-020-06493-6 32507918

[11] Yu W, Xu W, Jiang X, Liang D, Jian W. Risk factors for recollapse of the augmented vertebrae after percutaneous vertebral augmentation: a systematic review and meta-analysis. World Neurosurg. 2018;111:119–29. 10.1016/j.wneu.2017.12.019 29253703

[12] Zhang L, Zou J, Gan M, Shi J, Li J, Yang H. Treatment of thoracolumbar burst fractures: short-segment pedicle instrumentation versus kyphoplasty. Acta Orthop Belg. 2013;79(6):718–25. 24563980

[13] Muto M, Marcia S, Guarnieri G, Pereira V. Assisted techniques for vertebral cementoplasty: why should we do it? Eur J Radiol. 2015;84(5):783–8. 10.1016/j.ejrad.2014.04.002 24801264

[14] Wang JN, Xie W, Song DW, Zou J, Yan Q, Feng T, et al. Recurrence of local kyphosis after percutaneous kyphoplasty: the neglected injury of the disc-endplate complex. Clin Interv Aging. 2023;18:827–34. 10.2147/CIA.S410992 PMC10202700 37229150

[15] Tsujio T, Nakamura H, Terai H, Hoshino M, Namikawa T, Matsumura A, et al. Characteristic radiographic or magnetic resonance images of fresh osteoporotic vertebral fractures predicting potential risk for nonunion: a prospective multicenter study. Spine (Phila Pa 1976). 2011;36(15):1229–35. 10.1097/BRS.0b013e3181f29e8d 21217433

[16] Omi H, Yokoyama T, Ono A, Numasawa T, Wada K, Fujisawa Y. Can MRI predict subsequent pseudarthrosis resulting from osteoporotic thoracolumbar vertebral fractures? Eur Spine J. 2014;23(12):2705–10. 10.1007/s00586-014-3490-9 25082761

[17] Lee KY, Kim MW, Seok SY, Kim DR, Im CS. The relationship between superior disc-endplate complex injury and correction loss in young adult patients with thoracolumbar stable burst fracture. Clin Orthop Surg. 2017;9(4):465–71. 10.4055/cios.2017.9.4.465 PMC5705305 29201299

[18] Fujiwara T, Akeda K, Yamada J, Kondo T, Sudo A. Endplate and intervertebral disc injuries in acute and single level osteoporotic vertebral fractures: is there any association with the process of bone healing? BMC Musculoskelet Disord. 2019;20(1):336. 10.1186/s12891-019-2719-5 PMC6642561 31324243

[19] Shafshak TS, Elnemr R. The visual analogue scale versus numerical rating scale in measuring pain severity and predicting disability in low back pain. J Clin Rheumatol. 2021;27(7):282–5. 10.1097/RHU.0000000000001320 31985722

[20] Arpinar VE, Gliedt JA, King JA, Maiman DJ, Muftuler LT. Oswestry disability index scores correlate with MRI measurements in degenerating intervertebral discs and endplates. Eur J Pain. 2020;24(2):346–53. 10.1002/ejp.1490 31595564

[21] Zhang A, Lin Y, Kong M, Chen J, Gao W, Fan J, et al. A nomogram for predicting the risk of new vertebral compression fracture after percutaneous kyphoplasty. Eur J Med Res. 2023;28(1):280. 10.1186/s40001-023-01235-y PMC10416413 37563667

[22] Chou KN, Lin BJ, Wu YC, Liu MY, Hueng DY. Progressive kyphosis after vertebroplasty in osteoporotic vertebral compression fracture. Spine (Phila Pa 1976). 2014;39(1):68–73. 10.1097/BRS.0000000000000042 24108287

[23] Yu CW, Hsu CY, Shih TT, Chen BB, Fu CJ. Vertebral osteonecrosis: MR imaging findings and related changes on adjacent levels. AJNR Am J Neuroradiol. 2007;28(1):42–7. PMC8134120 17213422

[24] Goldstein S, Smorgick Y, Mirovsky Y, Anekstein Y, Blecher R, Tal S. Clinical and radiological factors affecting progressive collapse of acute osteoporotic compression spinal fractures. J Clin Neurosci. 2016;31:122–6. 10.1016/j.jocn.2016.02.020 27387197

[25] Chinese Medical Association, Committee of Osteoporosis and Bone Mineral Diseases. Guidelines for the diagnosis and treatment of primary osteoporosis. Chin Gen Pract. 2017;20(32):3963–82.

